# Supplementary material for: Peroxisomal ROS control cytosolic Mycobacterium tuberculosis replication in human macrophages
Source: J Cell Biol. 2023 Sep 22;222(12):e202303066. doi: 10.1083/jcb.202303066 (PMC10515436; doi:10.1083/jcb.202303066)
Supplement: Table S1 — shows cloning primers and oligos sequences used in this study. [file JCB_202303066_TableS1.docx]

**Table S1. Cloning primers and oligos sequences used in this study**

| **Primer Name** | **Sequence 5’->3’** |
| --- | --- |
| Fw_Hyper_BamHI | atacgggatccATGGAGGAGATGGCAAGCCAGCAGG |
| Rv_Hyper_PTS1_NotI | agtatagcggccgcctacagcttggaAACCGCCTGTTTTAAAAC |
| Top PST1 | GTACAAGTCCAAGCTGTAG |
| Bottom PST1 | GGCCCTACAGCTTGGACTT |
| PEX3_cDNA_F_infusion | CGTCAGATCCGCTAGCCATGCTGAGGTCTGTATG |
| PEX3_cDNA_R_TURBO_infusion | CATGGTGGCGACCGGTCCAAGTTTCTCCAGTTGCTGAG |
