# Supplementary material for: Peroxisomal ROS control cytosolic Mycobacterium tuberculosis replication in human macrophages
Source: J Cell Biol. 2023 Sep 22;222(12):e202303066. doi: 10.1083/jcb.202303066 (PMC10515436; doi:10.1083/jcb.202303066)
Supplement: Table S2 — shows guide RNAs and their sequences used in this study. [file JCB_202303066_TableS2.docx]

**Table S2. Guide RNAs and their sequences used in this study.**

| **Name sgRNAs** | **ID (WGE)** | **Sequence 5’->3’** |
| --- | --- | --- |
| CR1_PEX3 | 1016380307 | TAAAATCATGAACCATCCTA |
| CR2_PEX3 | 1016380317 | ATCCACCAATTATGTTTAAC |
| CR4_PEX3 | 1016380350 | GAAACCAGTGACTTGACAAA |
| CR3_PEX3 | 1016380352 | ATAAAATCAGATACCATCCT |
| CR6_PEX3_pool | 1016380338 | TCAAGTATTCAGCACCTACT |
| CR5_PEX3_pool | 1016380345 | TGACAGTGATCAATTCTGTC |
| CR7_PEX3_pool | 1016380311 | TGTATACAGTACCTGTATGC |
| CR1_CYBB_pool | 1189063726 | CTTCTAGTCAGCACTGGCAC |
| CR2_CYBB_pool | 1189063741 | TTACCGCACTGGAACCCCTG |
| CR3_CYBB_pool | 1189064446 | GAGTTCGAAGACAACTGGAC |
| CR4_CYBB_pool | 1189064450 | CCTTTCATAAAATGGTGGCA |
