# Supplementary material for: Peroxisomal ROS control cytosolic Mycobacterium tuberculosis replication in human macrophages
Source: J Cell Biol. 2023 Sep 22;222(12):e202303066. doi: 10.1083/jcb.202303066 (PMC10515436; doi:10.1083/jcb.202303066)
Supplement: Table S3 — shows primers and primer sequences used in this study. [file JCB_202303066_TableS3.docx]

**Table S3. Primers and primer sequences used in this study.**

| **Primer Name** | **Sequence 5’->3’** |
| --- | --- |
| PEX3_GF1 | CTACAATTCTTTGTGCTTCCCAGAC |
| PEX3_GR2 | GCCATTACTACAGAGCCAAGAAAAG |
| PEX3_SF1 | CACCTAATATATTTCATTGG |
