# Supplementary material for: Peroxisomal ROS control cytosolic Mycobacterium tuberculosis replication in human macrophages
Source: J Cell Biol. 2023 Sep 22;222(12):e202303066. doi: 10.1083/jcb.202303066 (PMC10515436; doi:10.1083/jcb.202303066)
Supplement: Table S4 — shows human qPCR primers used in this study. [file JCB_202303066_TableS4.docx]

**Table S4. Human qPCR primers used in this study.**

| **Gene name** | **Forward primer sequence**  **(5’ to 3’)** | **Reverse primer sequence**  **(5’ to 3’)** |
| --- | --- | --- |
| *PEX11α* | GAGAAGGTGGTAATGAAGCTCAA | GCTCTGCTCAGTTGCCTGT |
| *ABCD3* | GCCTGCACGGTAAGAAAAGTG | AGCCTTGAGAAAAACACCTTGTC |
| *PEX3* | GGCTGAGTTCTTTCGACCTA | ACTGCAAACTGAATGGATCTGTC |
| *PMP34* | CTTCGACTTCAGGTTGATGAGA | TGACCTTTGACCCAGAGTGC |
| *CROT* | GTGGTGGCTGAATGTTGCCTA | TTGGAGGCCAGTAGTGTTCAA |
| *EHHADH* | GGTCAACGCGATCAGTACGAC | CCTCTGCTCCACAAATCACAATG |
| *AGPS* | TTAGTGGCATGGGTTTACCAAC | CTCGATCATCTGCCTCTTGTG |
| *FAR1* | AGACACCACAAGAGCGAGTG | CCAGTTTAGGTTGGGTGAGTTC |
| *ACOX1b* | GGGCCTCAATTACTCCATGTTT | TGGGCGTAGGTGCCAATTATC |
| *GNPAT* | GAGGAGGCATGTCAGTGACTT | ACAAAACCGAATGGCTCCAAG |
| *PEX11β* | CGCCCAGTATGCTTGCTCTC | TCGATTGAGGTGACTAACAGTGA |
| *PPARα* | ACACTGTGTATGGCTGAGAAGA | GACGGTCTCCACTGACGTG |
| *ACOX1a* | CCTGAACGACCCAGACTTCC | TGCCTGGTGAAGCAAGGTG |
| *PEX5* | AAGCCTTTGGGAGTAGCTTCT | GGACACAAGGGGTGCATTC |
| *PPARγ1* | AAAGAAGCCAACACTAAACC | CTTCCATTACGGAGAGATCC |
| *ACAA1* | GCGGTTCTCAAGGACGTGAAT | GTCTCCGGGATGTCACTCAGA |
| *CRAT* | GTGGCTCAAGACCGCCTAC | GCAGCAAATCGGAGCTGAC |
| *PGC1β* | GATGCCAGCGACTTTGACTC | ACCCACGTCATCTTCAGGGA |
| *PEX14* | GCCACGGCAGTGAAGTTTCTA | TGCTGGAAGGCCATATCAATCT |
| *PEX16* | GTGCGGGGCTTCAGTTACC | GGTTAGAGGCAGAGTACACCA |
| *PEX11γ* | GGGGACACGTCTGTTGGTG | ACAGGGGTAGTAGAGCTGGTC |
| *ABCD1* | GCTGGCATGAACCGGGTATT | GCCACATACACCGACAGGAA |
