# Supplementary figures and images for: Peroxisomal ROS control cytosolic Mycobacterium tuberculosis replication in human macrophages
Source: J Cell Biol. 2023 Sep 22;222(12):e202303066. doi: 10.1083/jcb.202303066 (PMC10515436; doi:10.1083/jcb.202303066)

SourceData F1

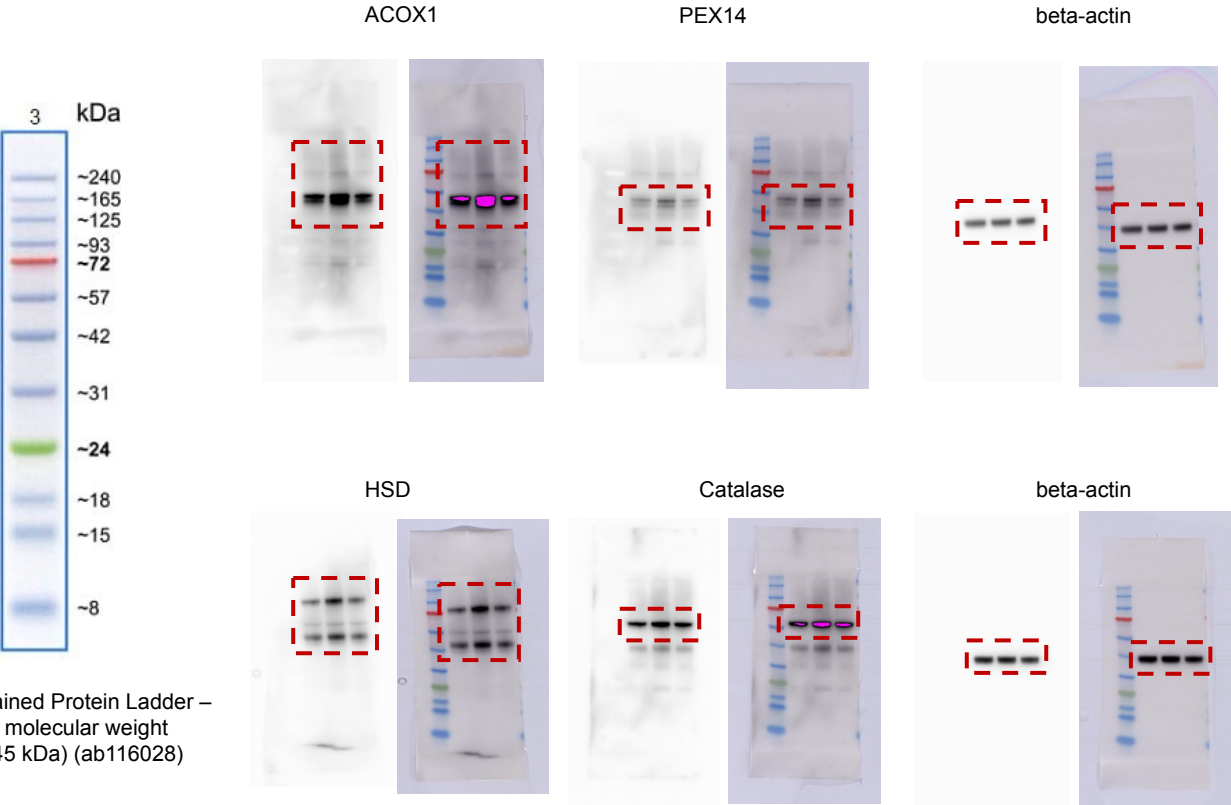

Supplement: SourceData F1 — is the source file for Fig. 1. [file JCB_202303066_SourceDataF1.pdf]

SourceData SF3

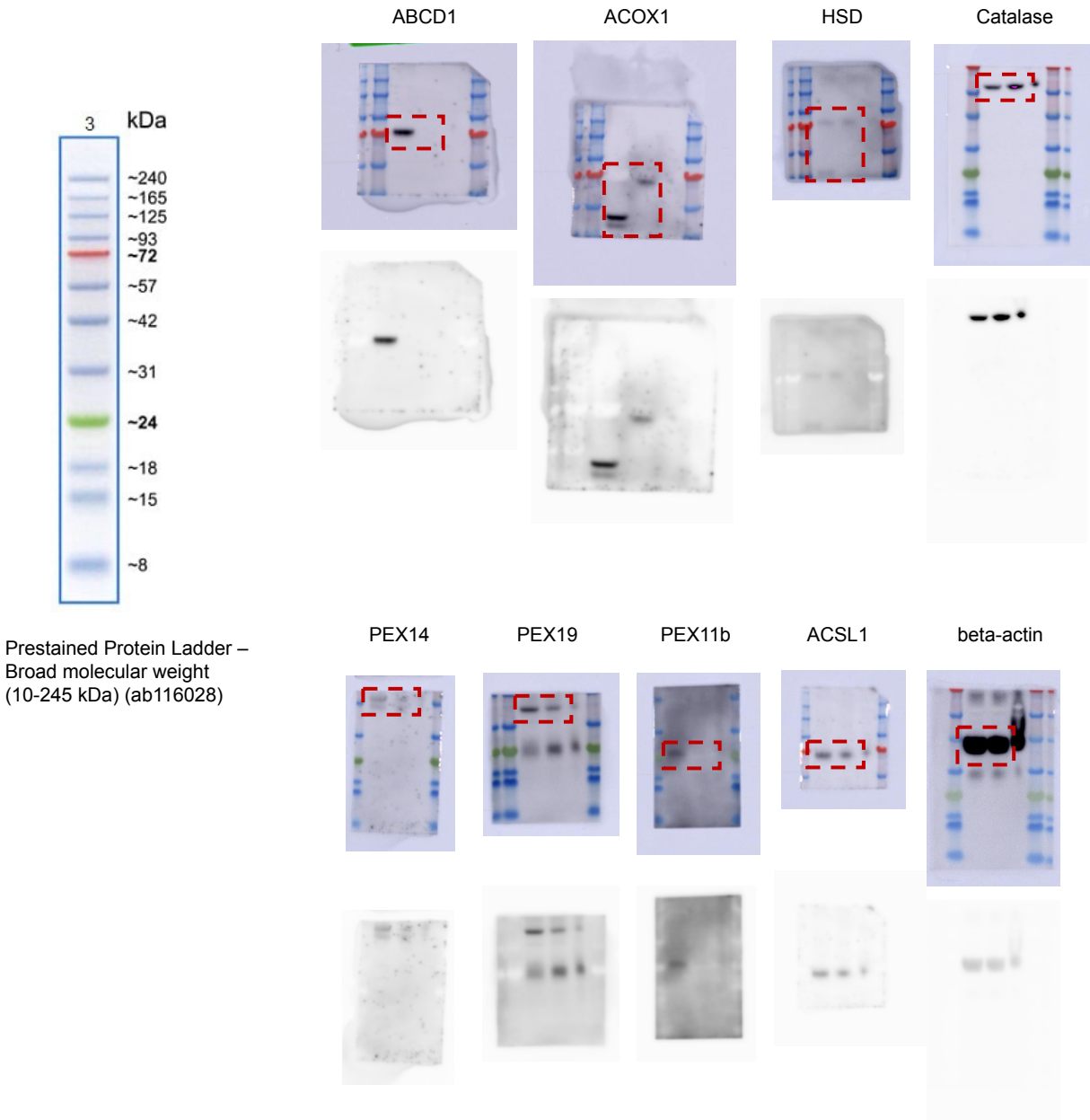

Supplement: SourceData FS2 — is the source file for Fig. S2. [file JCB_202303066_SourceDataFS2.pdf]
